# Effects of the Mg/Si Ratio on Microstructure, Mechanical Properties, and Precipitation Behavior of Al–Mg–Si–1.0 wt %-Zn Alloys

**DOI:** 10.3390/ma11122591

**Published:** 2018-12-19

**Authors:** Yong Li, Guanjun Gao, Zhaodong Wang, Hongshuang Di, Jiadong Li, Guangming Xu

**Affiliations:** State Key Laboratory of Rolling and Automation, Northeastern University, Shenyang 110819, China; liyong.neu@163.com (Y.L.); zhaodongwang@263.net (Z.W.); dhshuang@mail.neu.edu.cn (H.D.); lijd@ral.nue.edu.cn (J.L.); xu_gm@epm.neu.edu.cn (G.X.)

**Keywords:** Al–Mg–Si–1.0 wt %-Zn alloy, Mg/Si ratio, recrystallization, texture, deep drawability, paint-bake response

## Abstract

Aluminum alloys are widely used as first-choice materials for lightweight automotive applications. It is important that an alloy have a balance between strength and formability. In this study, the alloys were melted, cast, hot rolled, and cold rolled into 1 mm-thick sheets. The microstructure, mechanical properties, and precipitation behavior of Al–Mg–Si–1.0 wt %-Zn alloys with Mg/Si ratios of 0.5, 1, and 2 after solution treatment were studied using optical and electron microscopy, a tensile test, the Vickers hardness test, and differential scanning calorimetry. The results showed that a high density and number of Al–Fe–Si particles were observed in the matrix, thus causing the formation of more homogeneous and smaller recrystallized grains after treatment with the solution. In addition, a higher volume fraction of cube_ND_ and P-types texture components formed during solution treatment. Also, a high *r* value and excellent deep drawability were achieved in the medium-Mg/Si-ratio alloy. The formation of denser strengthening precipitates led to a better paint-bake hardening effect in comparison with the other two alloys. Furthermore, the precipitation kinetics were enhanced by the addition of Si, and the addition of Zn did not alter the precipitation sequence of the Al–Mg–Si alloy. The dual-phase strengthening effect was not achieved in the studied alloys during paint-bake treatment at 175 °C.

## 1. Introduction

Precipitation hardening of an Al–Mg–Si alloy is an ideal choice for the automobile industry because of its high strength-to-weight ratio, good formability, excellent corrosion resistance, and low cost [1,2,3]. Increased strength in alloys is acquired by paint-bake hardening after the solution is heat-treated at 560 °C and rapidly quenched to room temperature (RT) [4,5,6]. During paint-bake hardening, a large number of transition phases are formed in the matrix, resulting in a strengthening effect. It is reported that the generally accepted precipitation sequence of Al–Mg–Si alloys during artificial aging is as follows [7,8,9,10,11]: SSSS → solute clusters → GP (Guinier Preston) zones/pre-β″ → β″ → β′, B′, U1, U2 → β phase → Si particles, where SSSS denotes the supersaturated solid solution. The GP zones are completely coherent with the matrix and are initially formed by the aggregation of Mg and Si atoms. The precipitate is semi-coherent with the Al matrix and contains less Si atoms than the GP zone [8]. Most of the rod-shaped U1, U2, and B′ phases form together with the rod-like β′ during over-ageing, which results in less strength increments [9]. The equilibrium phase β is FCC (Face center cubic) in structure. It forms in plates with the composition Mg_2_Si. Previous studies have shown that the semi-coherent, needle-like β″ phase is the main hardening precipitates in Al–Mg–Si alloys during paint-bake hardening [12].

Mg and Si are the main alloying elements in Al–Mg–Si alloys. The β″ phase forms with enhanced yield strength during the paint-bake hardening process. Most of the Mg-rich clusters are formed as a result of the segregation of high-Mg atoms, while high-Si atoms result in the formation of dispersed Si-rich clusters. Spherical Si particles have a minimal effect on the conductive behavior of the alloys [13,14]. An important research area is the study of the effects of additional alloying elements on Al–Mg–Si alloys, because they directly affect the microstructures, as well as the precipitate structures, during heat treatment [15]. Generally, Zn and Mg atoms form the η′-MgZn_2_ hardening phase in Al–Zn–Mg alloys, leading to a strong precipitation-strengthening effect. In addition, Zn is a trace element in aluminum scrap metal from the recycling process [11,16,17]. Therefore, Zn-containing Al–Mg–Si alloys are thought to possess a potential bake-hardening response with an acceptable formability.

In current applications of Al–Mg–Si alloys, different Mg/Si ratios have been extensively studied. However, several views still remain controversial. For example, the effects of alloy composition on the microstructure and formability of Zn-containing Al–Mg–Si alloys have yet to be fully explored. It is not known whether the addition of Zn affects the precipitation behavior of Al–Mg–Si alloys during an artificial aging treatment. In this study, the effects of adding 1.0 wt % Zn into an Al–Mg–Si alloy and changing the ratio of Mg/Si during the age-hardening strengthening phases during paint-bake treatment were studied. The influences of the Mg/Si ratio on the recrystallization behavior during solid solution treatment and the mechanical properties after pre-aging treatment were also investigated. The aim of the present work was to find the optimum composition of an Al–Mg–Si alloy with uniform microstructure, good formability, and rapid aging response and to provide technical support and data references for the composition design of new alloys and the selection of related heat treatments for in situ production.

## 2. Materials and Methods

The compositions of the three tested groups of alloys are presented in Table 1. The total weight percentages of Mg and Si (Mg + Si) were kept nearly identical for alloys A, B, and C. Three alloys with Zn content of 1.0 wt % were prepared with 99.7 wt % industrial pure Al and master alloys (Al–20 wt % Si, Al–20 wt %, Mg and Al–25 wt % Zn). The master alloys were first melted in a graphite crucible and then poured into copper molds and water cooled at 720 °C. The composition of the studied alloys was independently determined using inductively coupled plasma atomic emissive spectrometry (ICP-AES, SPECTRO Analytical Instruments GmbH, Boschstr, Kleve, Germany). After face milling, all ingots with nominal dimensions of 210 mm × 110 mm × 25 mm were homogenized in an air circulation furnace at 470 °C for 5 h and then at 540 °C for 16 h. The hot rolling schedule was 25.0 → 21.0 → 16.0 → 11.0 → 7.5 → 6.0 (mm), with the beginning and finishing temperatures of 500 °C and 350 °C, respectively. In the cold rolling process, the reduction schedule was 6.0 → 3.9 → 2.6 → 1.8 → 1.3 → 1.0 (mm).

**Figure 1:**
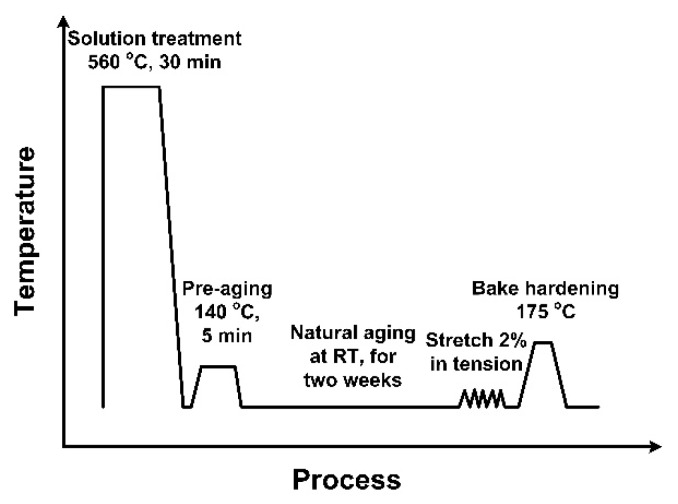
Schematic representation of the heat treatment process of aluminum alloy sheets.

All sheets were completely recrystallized and solution-treated in an air circulation furnace at 560 °C for 30 min followed by quenching to RT. Then, the sheets were immediately pre-aged at 140 °C for 5 min after solution treatment. Finally, these sheets were kept at RT for two weeks (T4P state). The stamping process was simulated with 2% deformation on a tensile tester. Furthermore, the alloys were artificially aged at 175 °C up to 16 h in an air circulation furnace for the paint-bake treatment. A schematic representation of the heat treatment procedure is illustrated in Figure 1.

A number of traditional testing methods were chosen for this study. According to the results obtained with these methods, reference data are provided for practical industrial production. The artificial aging behavior of the alloys was studied using a Vickers hardness tester (KB3000BVRZ-SA, KB Prüftechnik GmbH, Im Weichlingsgarten, Hochdorf-Assenheim, Germany) with a load of 49 N and a dwell time of 10 s to study the artificial aging behavior of the alloys. To reduce the error, five indentations were made to obtain the average hardness value of each alloy. The hardness values were then measured. A tensile test was performed at RT using an INSTRON-4206 electronic universal testing machine (Instron Corporation, Norwood, MA, USA) with a load speed of 3 mm/min. The microstructure of the alloys was observed using the Imager M2m ZEISS metallurgical microscope (ZEISS, Carl-Zeiss-Straße, Oberkochen, Germany). The specimens used for recrystallization microstructure observation were mechanically grounded and electropolished in a 10 vol % perchloric acid alcohol solution at 25 V for 30 s. Then, the electropolished specimens were anodized using a solution consisting of 43 vol % phosphoric acid + 38 vol % sulfuric acid + 19 vol % distilled water at 20 V for 2 min. The surface morphologies were characterized using a ZEISS ULTRA 55 field emission scanning electron microscope (SEM) (ZEISS, Carl-Zeiss-Straße, Oberkochen, Germany) that was equipped with electron backscatter diffraction. The precipitates formed during artificial aging were observed using a Tecnai G2 F20 transmission electron microscope (TEM) (FEI Company, Hillsboro, OR, USA) at 200 kV operating voltage. The TEM specimens were prepared using a TenuPol-5 jet-polisher at an operating voltage of 15 V, and a 30 vol % nitric acid in methanol solution (stored between −25 °C and −30 °C) was used as the electrolyte. The TEM bright-field images revealed that the Al matrix was aligned in the <001> direction in all the tested alloys. Differential scanning calorimetry (DSC) was conducted using a Q100 system (with a heating rate of 10 °C/min) under an argon atmosphere in the temperature range of 30 °C to 400 °C. The specimens for DSC were cleaned using an ultrasonic cleaning machine, and an empty pure aluminum crucible was used as the reference material.

## 3. Results and Discussion

### 3.1. Microstructures

**Figure 2:**
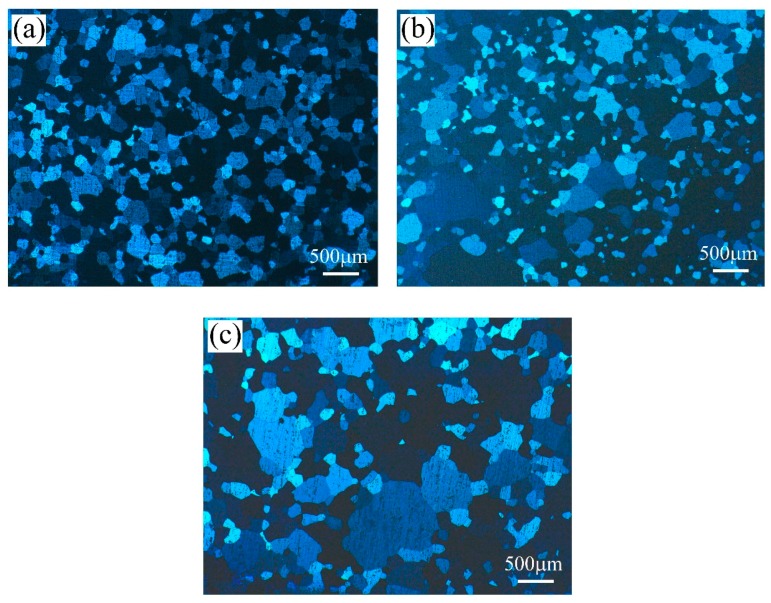
Recrystallized grain structures after solution treatment: (**a**) alloy A; (**b**) alloy B; and (**c**) alloy C.

Figure 2 displays the recrystallized grains of the three groups of alloys after the solution treatment. The medium Mg/Si alloy (alloy A) exhibited smaller and narrowly distributed recrystallized grain sizes of 185 μm. In contrast, the excess Mg alloy (alloy B) and the excess Si alloy (alloy C) developed coarse and inhomogeneous recrystallized grains after solution treatment. The average grain sizes of alloys B and C were found to be 203 and 259 μm, respectively.

**Figure 3:**
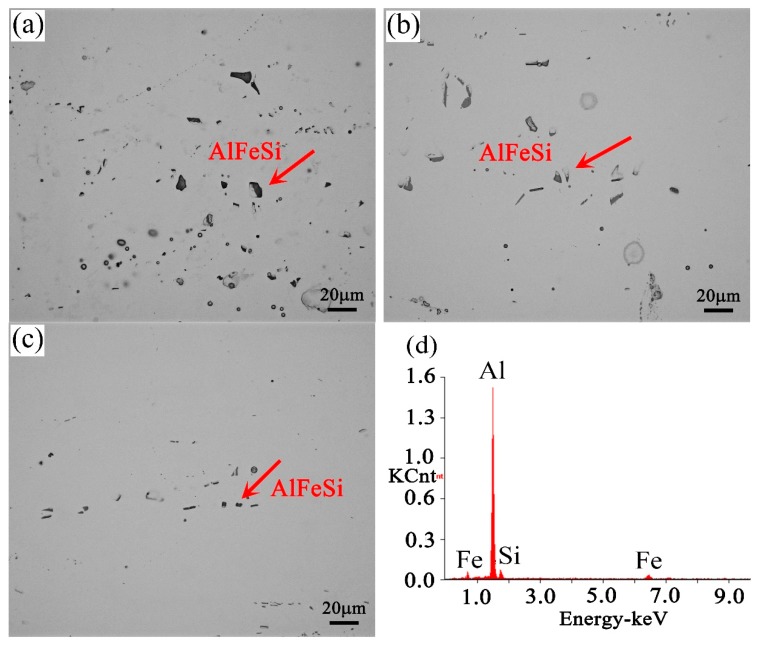
SEM images of particle distribution in the solution-treated alloys: (**a**) alloy A; (**b**) alloy B; (**c**) alloy C, and (**d**) EDS spectra of the particles.

The SEM images showed the distribution of the particles (red arrow) in the alloys, as shown in Figure 3. It was observed that alloy A caused a homogeneous particle distribution; the density of the particles decreased in alloys B and C. It was also discernible that some particles existed at the grain boundary (GB), and most particles were distributed in the matrix. Energy dispersive X-ray analysis (EDS) revealed that the particles contained Al, Si, and Fe. It is proposed that the particles most likely consisted of stable α-Al–Fe–Si phases, which were often observed in the Al–Mg–Si alloy [18,19,20].

The recrystallized grains first nucleated and grew at a position of high-energy fluctuation (dislocation, large particles, and so on) during solution treatment. The number and distribution of the large particles had a great influence on the recrystallization grains [21]. In this study, the homogeneous and high-density large Al–Fe–Si particles in alloy A acted as nucleation sites for the recrystallized grains, thus causing the formation of smaller grains during solution treatment. However, in alloys B and C, the number of large Al–Fe–Si particles was relatively low, and the distribution was not uniform, which led to the uneven nucleation of recrystallized grains (Figure 3). In addition, because the recrystallization of the alloy was completed before the soluble particles re-dissolved into the matrix, it was inferred that compared to alloys B and C, more soluble small particles existed in alloy A before solution treatment. These small particles exerted a Zener drag and effectively retarded the migration of grain boundaries, which led to the formation of uniform and small recrystallized grains [21]. 

### 3.2. Deep Drawability Analysis

**Figure 4:**
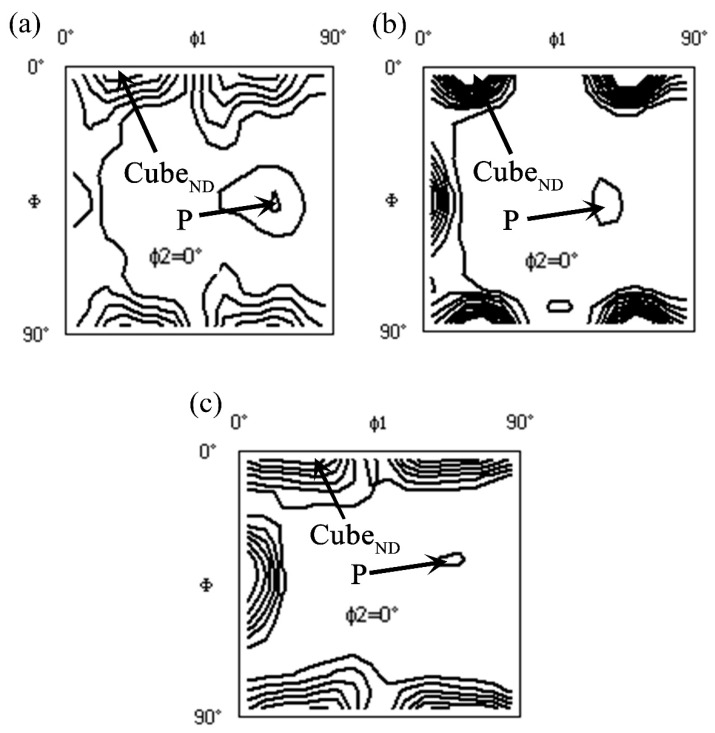
Recrystallization textures in the solution-treated alloys: (**a**) alloy A; (**b**) alloy B; and (**c**) alloy C.

Figure 4 shows the recrystallization textures in the three groups of alloys after solution treatment. Alloy A displayed Cube_ND_ and P orientations with intensities of 5.61 and 2.09, respectively. The volume fraction of the P component was 9.71%. In contrast, the B and C alloys consisted of lower intensities and smaller volume fractions of the P component (Table 2). Accordingly, the density and distribution of the large Al–Fe–Si particles affected not only the recrystallized grains but also the texture components and intensities.

**Figure 5:**
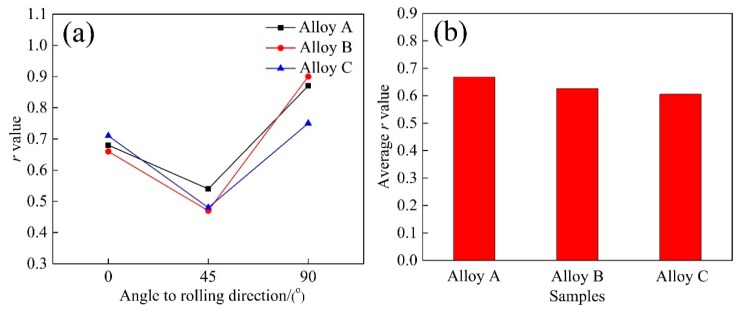
(**a**) Values of *r* in three different directions; (**b**) the calculated average *r* values of alloys A, B, and C.

The *r* values of the three groups of alloys were calculated using a 15% deformation in three different directions, as shown in Figure 5. It was obvious that the *r* value of alloy A was higher than those of the other two alloys at a 45° direction. At a 90° direction, alloy C manifested the lowest *r* value. The average *r* value (Figure 5b) was calculated using

(1) $$\bar{r}=\frac{r_{0^{{}^{\circ}}}+2r_{45^{{}^{\circ}}}+r_{90^{{}^{\circ}}}}{4},$$

where $r_{0^{{}^{\circ}}}$, $r_{45^{{}^{\circ}}}$, and $r_{90^{{}^{\circ}}}$ are the *r* values in three different directions. The result revealed that the average *r* value decreased from alloy A to alloy C. Therefore, the alloy A possessed relatively better deep drawability.

The texture components significantly affected the *r* values and the cube_ND_ and P components, and these were favorable for deep drawability of the Al–Mg–Si alloys. It was reported that the stimulated *r* values for the cube and the P component were 0.5 and 2.8, respectively [22,23,24]. Liu et al. [25] posited that the cube_ND_ component resulted in a higher *r* value (>0.5) than the cube component. Moreover, when a certain number of coarse particles (>1 μm) existed in the matrix, a particle stimulated nucleation (PSN) response occurred in the alloys during solution treatment. In alloy A, homogeneous and high-density large Al–Fe–Si particles were observed in the matrix, and recrystallization nuclei formed around these large particles. This process resulted in the increment of the cube_ND_ and P components. In contrast, a nonuniform distribution of low-density large Al–Fe–Si particles formed in both the B and C alloys, causing a decrease in the volume fraction of the cube_ND_ and P components. Although a large number of clusters formed in the matrix of the three alloys after pre-aging, this had little effect on the deep drawability. The grain morphology also profoundly influenced the *r* values. The small and uniform recrystallized grains contributed to a higher *r* value [24]. Hence, the alloy A achieved improved deep drawability.

### 3.3. Simulated Paint-Bake Cycle and Precipitation-Hardening Behavior

**Figure 6:**
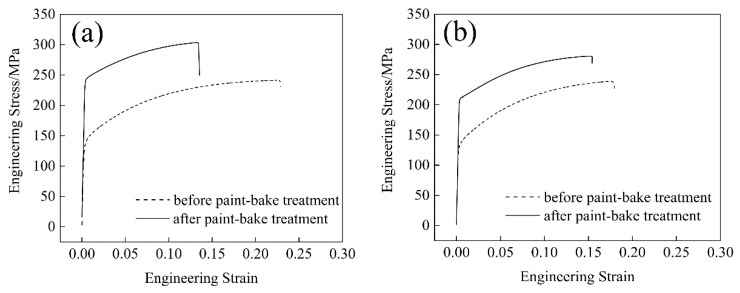

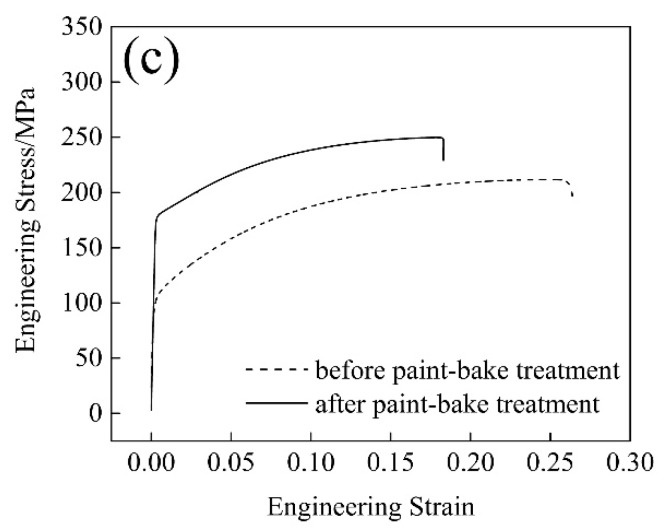
Engineering stress–strain curves of the alloys before and after the paint-bake treatment at 185 °C for 30 min: (**a**) alloy A; (**b**) alloy B; and (**c**) alloy C.

**Figure 7:**
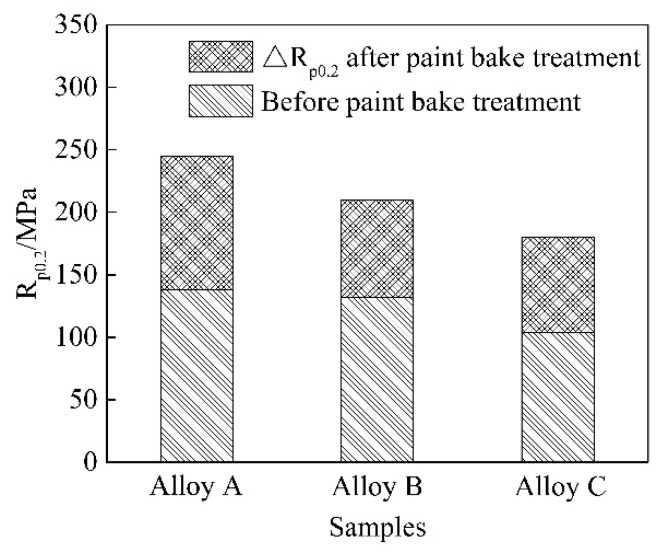
Increment of yield strength before and after the simulated paint-bake treatment.

Figure 6 shows the engineering stress strain curves of the three groups of the alloys before 2% deformation and after the paint-bake treatment at 185 °C for 30 min. The increments of yield strengths, ΔRp_0.2_, before and after the paint-bake treatment were measured (Figure 7). Before 2% deformation (T4P state), the yield strengths of alloys A and B were similar, whereas alloy C manifested a lower value. After the simulated paint-bake treatment, the △Rp_0.2_ value of alloy A was higher than those of the other two alloys, as shown in Figure 7.

A clustering of supersaturated solute atoms occurred in the matrix during the pre-aging treatment at 140 °C for 5 min. Before the paint-bake treatment, the strength of the alloys depended on the number of clusters and the recrystallized grain morphology. Pre-aging was a short-term heat-treatment process that occurred at a low temperature, resulting in a small number of clusters that formed in the matrix [26]. The recrystallization microstructure revealed an increase in the size of the recrystallized grain in alloys A and C. Alloy C exhibited relatively larger grains. Under a similar applied stress, the stress concentration on the smaller grains was weak. Plastic deformation of the adjacent grains required a larger applied stress. The conventional Hall–Petch relationship is given by

(2) $$\sigma_{y}=\sigma_{o}+k_{y}d^{-1/2},$$

where $\sigma_{y}$ is the yield stress, $\sigma_{o}$ is the lattice friction stress, and $k_{y}$ is a constant of yielding. The smaller the grain size was, the higher the yield strength was [27]. However, the average grain size of the three groups of alloys was large (≥180 μm), and $k_{y}$ was very small for Al in this study. Therefore, the contribution of grain size to the increment of the alloy’s strength was not significant [28]. The clusters rapidly grew along one direction during the paint-bake treatment, and lattice distortion in the matrix was introduced by the grown precipitates. Consequently, the precipitate pinning forces on moving dislocations were strong and acted as effective obstacles for the dislocation movement. These factors eventually increased the strength of the alloys [12]. A high strength was achieved by the formation of strengthening precipitates and a sufficient supply of supersaturated solute Mg and Si atoms in alloy A. Coarse Al–Fe–Si particles existed in the matrix before and after paint-bake hardening. These coarse particles possessed poor deformation-coordination ability and became the source of cracks in the tensile deformation process of the alloy, which reduced the elongation of the alloy [29]. Hence, the elongation increased with a decrease in the number of Al–Fe–Si particles from alloy A to alloy C (Figure 6).

**Figure 8:**
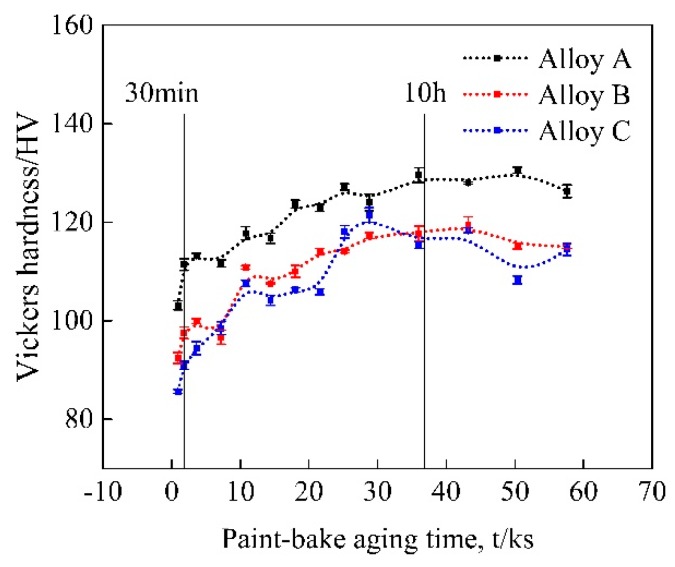
Vickers hardness curves of the alloys with paint-bake treatment at 175 °C for 16 h.

The Vickers hardness curves of the three groups of alloys with paint-bake treatment for 16 h are presented in Figure 8. The figure shows that the hardness of the alloys increased with prolonged aging time, and a nearly constant value was attained at 175 °C after 10 h of paint-baking treatment. The hardness of alloy A was higher than that of alloys B and C and increased rapidly within the first 30 min. This finding agrees with the result shown in Figure 7.

**Figure 9:**
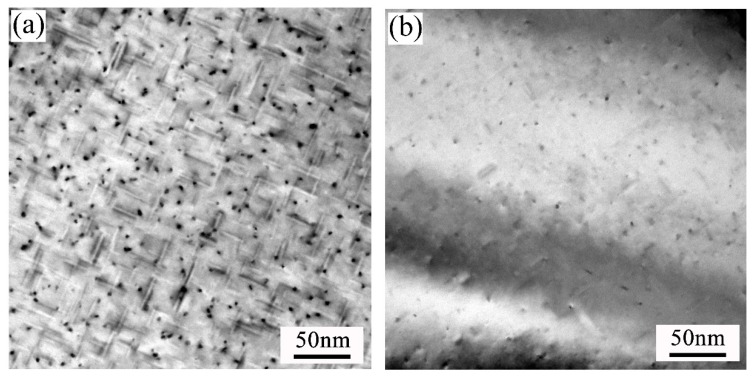

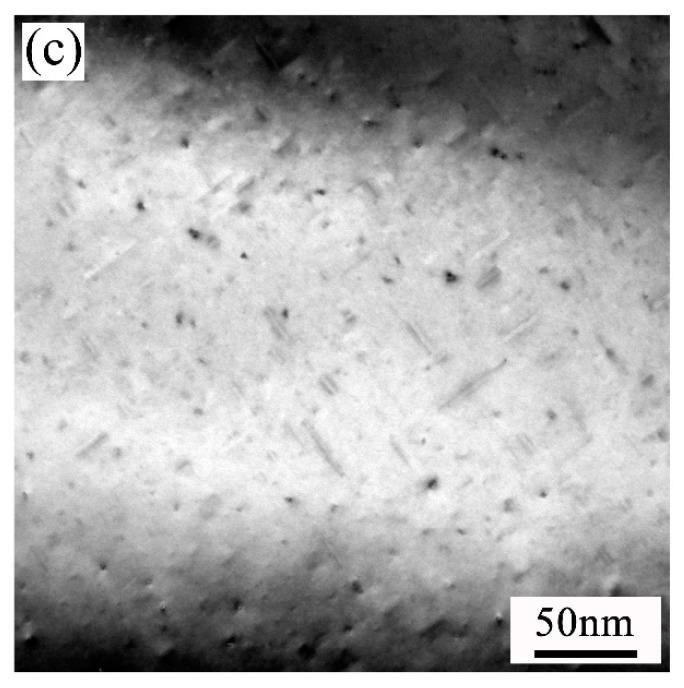
TEM images of the alloys after the paint-bake hardening treatment at 175 °C for 10 h: (**a**) alloy A; (**b**) alloy B; and (**c**) alloy C.

**Figure 10:**
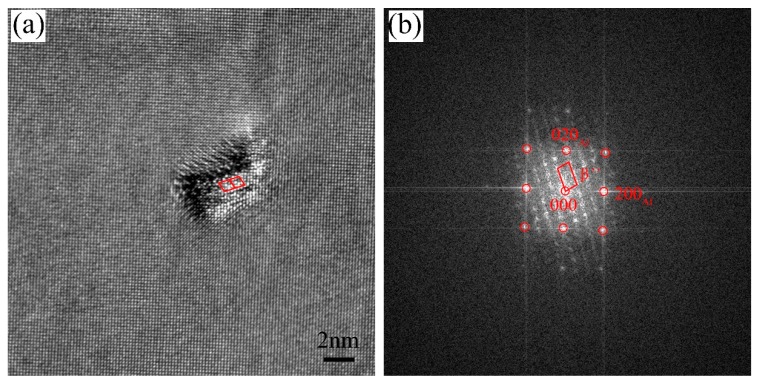
(**a**) HRTEM and (**b**) FFT patterns of the precipitates in the alloys after the paint-bake hardening treatment at 175 °C for 10 h.

Figure 9 displays the bright-field TEM images of the alloys after paint-bake hardening at 175 °C for 10 h. All images were acquired along the <100>_Al_ zone axis. In the three groups of alloys, high amounts of fine dot-like and needle-like precipitates were uniformly distributed in the Al matrix. The dot-like precipitates appeared needle-like when viewed end-on in another direction. In alloy A, a dense amount of strengthening precipitates were observed in the matrix, and most of the precipitates were larger than those in alloys B and C. This was why alloy A possessed the highest hardness among the three groups of alloys. The HRTEM and the corresponding fast Fourier transform (FFT) patterns of the precipitates are exhibited in Figure 10. The orientation relationships between the precipitates and the Al matrix were (010)_β″_//(001)_Al_, [100]_β″_//[320]_Al_, and [001]_β″_//[13̅0]_Al_. This observation is consistent with previous studies by Yang et al. [30]. On the basis of the HRTEM and FFT patterns, the precipitates observed were identified as the β″ phase, which was the main hardening precipitate in the Al–Mg–Si alloy system.

**Figure 11:**
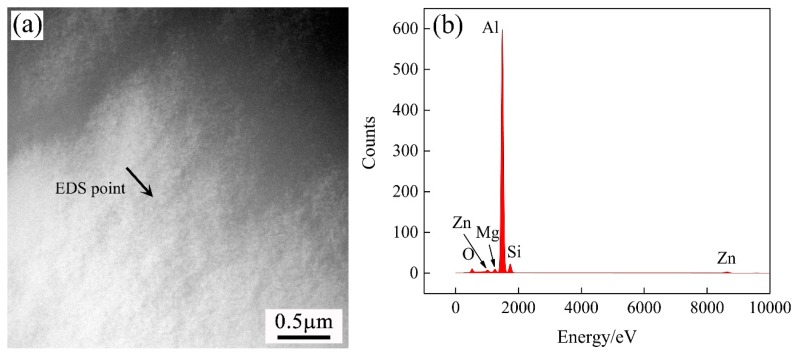
(**a**) The EDS measurement point in the matrix and (**b**) the EDS spectra of the measurement point.

High Zn (1.0 wt %) was added to the three groups of alloys. However, only β” precipitates were observed along the <001>_Al_ direction, while no plate-like precipitates were found on the {111}_Al_ plane, which was an effective strengthening phase for Al–Zn–Mg alloy system. This demonstrated that the η′ phase did not form in the studied alloys. It is worth noting that the normal aging temperature of the Al–Zn–Mg alloys was lower than 120 °C. Therefore, it was impossible that the η′ strengthening phase formed in the Al–Mg–Si alloy during paint-bake treatment at 175 °C [16]. The EDS analysis (Figure 11) revealed that most of the Zn atoms existed in the Al matrix, and the alloys were strengthened by the solution strengthening response. Therefore, no other precipitates formed during the paint-bake hardening at 175 °C, and the addition of Zn did not alter the precipitation sequence of the Al–Mg–Si alloy.

**Figure 12:**
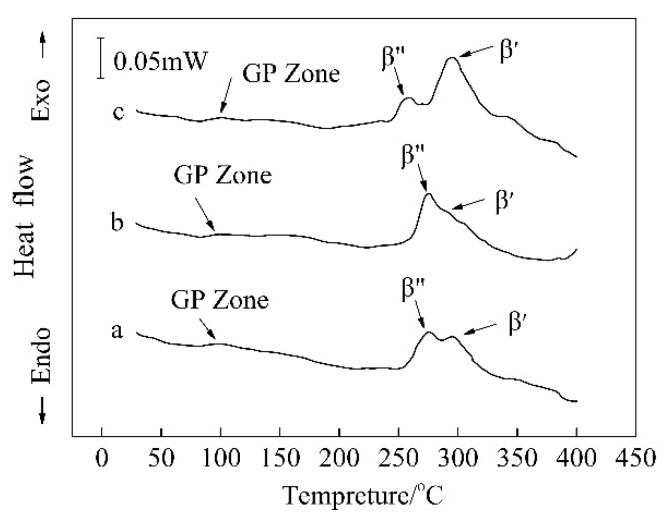
DSC flow curves of the (**a**) alloy A; (**b**) alloy B; and (**c**) alloy C after PA (Pre-aged) treatment at 140 °C for 5 min.

DSC analysis was conducted to investigate the effects of various Mg/Si ratios and Zn content on the precipitation sequence of the Al–Mg–Si alloy. Figure 12 shows the DSC curves of the three groups of alloys after PA (Pre-aging) treatment at 140 °C for 5 min. Three exothermic peaks that coincided with the precipitations of the GP zone, β″ phase, and β′ phase were observed in each alloy in the temperature range from 30 °C to 400 °C [31,32,33,34]. In alloys A and B, the exothermic peaks of the β″ phase appeared at ~275 °C. However, in alloy C, the peak of the β″ phase was observed at ~255 °C. Excess Si effectively increased the precipitation kinetics by changing the Mg/Si ratio in the initial clusters that formed during PA treatment. Additionally, excess Mg decreased the solubility of Mg_2_Si in the matrix, and the growth of Mg_2_Si phase consumed the supersaturated solute atoms, which led to a suppression of the precipitation kinetics of the β″ phase [35]. Hence, this indicated that Si could decrease the peak temperature of the β″ precipitate in the DSC curves. Generally, Zn and Mg formed the strengthening phases, η′ and η, in the Al–Zn–Mg alloy. The peaks at the temperatures of ~185 °C and ~230 °C were related to the η′ and η phases, respectively [11,16]. However, no obvious exothermic peaks existed at these temperatures in the DSC curves. The η′ or η phase did not form in the matrix during the artificial aging treatment. Hence, the peaks related to the precipitation or phase in the DSC curves also indicated that the addition of elemental Zn did not alter the precipitation sequence of the Al–Mg–Si alloy.

## 4. Conclusions

The influence of Zn content and different Mg/Si ratios on the microstructure, mechanical properties, and the precipitation behavior of Al–Mg–Si alloys was investigated in this study. The following inferences can be drawn from the results of this research.

1. The composition-optimized alloy was a medium-Mg/Si-ratio alloy. In this alloy, more homogeneous and smaller recrystallized microstructures combined with high *r* value and excellent deep drawability were developed in comparison with the other two alloys.
2. In the medium-Mg/Si-ratio alloy, the denser and larger strengthening precipitates contributed to a better paint-bake hardening response. Zn atoms existed in the matrix as supersaturated solute atoms and strengthened the alloys to a certain extent.
3. The addition of Zn did not affect the precipitation sequence of the Al–Mg–Si alloy. No other precipitates formed, and the dual-phase strengthening effect was not achieved in the three groups of alloys during paint-bake treatment at 175 °C.

## Figures and Tables

**Table 1:** Chemical composition (in wt %) of the tested alloys.

Alloy	Zn wt %	Mg wt %	Si wt %	Fe wt %	Mg + Si	Mg/Si	Comment
A	1.04	0.75	0.76	0.129	1.51	0.99	Medium Mg/Si
B	1.06	1.04	0.52	0.126	1.56	2.00	Excess Mg
C	1.03	0.51	1.03	0.128	1.54	0.50	Excess Si

**Table 2:** Volume fractions of recrystallization textures in the solution-treated alloys.

Alloy	Component	Intensity	Volume Fraction (%)
A	Cube_ND_	5.61	5.72
	P	2.09	9.71
B	Cube_ND_	13.12	9.82
	P	1.34	4.58
C	Cube_ND_	6.72	8.28
	P	1.16	3.11
